# Effect of powder air polishing and ultrasonic scaling on the marginal and internal interface (tooth-veneer) of ceramic veneers: an in vitro study

**DOI:** 10.1007/s00784-024-06046-x

**Published:** 2024-11-26

**Authors:** Florian Fuchs, Laura Antonia Mayer, Lena Unterschütz, Dirk Ziebolz, Nadia Oberueck, Ellen Schulz‑Kornas, Sebastian Hahnel, Andreas Koenig

**Affiliations:** 1 Department of Prosthodontics and Materials Science, Leipzig University, Liebigstraße 12, 04103 Leipzig, Germany; 2 Department of Cariology, Endodontology and Periodontology, Leipzig University, Liebigstraße 12, 04103 Leipzig, Germany; 3 Department of Prosthetic Dentistry, UKR University Hospital Regensburg, Franz‑Josef‑Strauß‑Allee 11, 93053 Regensburg, Germany

**Keywords:** Adhesive, Surface texture, Prophylaxis, Dental cleansing, Artificial accelerated aging, Thermal cycling

## Abstract

**Objectives:**

This study investigated the influence of prophylactic treatments and thermocycling on the marginal and internal veneering interface (tooth-veneer) as well as on the surface texture of ceramic veneers.

**Materials and methods:**

A total of 32 extracted human premolars were restored with veneers made of lithium disilicate (LDS) or zirconia-reinforced lithium silicate (ZLS). An artificial aging of the specimens was conducted via five cycles of both thermocycling (5/55°C) and prophylactic treatment (powder air polishing or ultrasonic scaling). Changes in the marginal interface and in the surface texture were examined using confocal laser scanning microscopy. The internal interface and the microstructure were investigated using micro X-ray computed tomography.

**Results:**

Artificial aging resulted in a deepening of the marginal interface across all groups (mean height: 4.51–15.74 μm, maximum height: 10.42–22.71 μm, cross-section: 256.68–1525.84 μm², regardless of the veneer material or prophylaxis method. No change in surface texture was observed. The internal interface exhibited defects for all groups after artificial aging. ZLS showed cracks in five out of eight veneers after exposure to ultrasonic scaling and thermocycling.

**Conclusion:**

Ceramic veneers exhibited a high resistance to prophylactic measures in terms of surface durability, but a deepening of the marginal interface should be taken into account. With regard to the formation of cracks within the material, the use of ultrasonic scaling is not recommended for ZLS veneers.

**Clinical relevance:**

The influence of artificial aging, including prophylactic treatments, plays a critical role in assessing longevity for veneers in defect-oriented and esthetic dentistry.

**Supplementary Information:**

The online version contains supplementary material available at 10.1007/s00784-024-06046-x.

## Introduction

Veneers represent a defect-oriented dental restoration form which is used in patients with tooth structure loss due to trauma or abrasion as well as for esthetic corrections of such as malformations [1, 2]. This minimally invasive treatment option has become feasible not least due continuous improvements in adhesive technology and the further development of digitalization [3]. In this context, computer-aided design/computer-aided manufacturing (CAD/CAM) technology represents an approach for reducing the time, cost, and laboratory equipment required for the fabrication of monolithic restorations [4–6]. This has also increased the choice of materials with sufficient esthetic and improved mechanical properties [7, 8]. Ceramics, which are known for their favorable optical properties with a wide range of shades and translucencies and improved flexural strength, are particularly suitable for the fabrication of veneers [9, 10].

Lithium disilicate ceramics (LDS), like the commonly used dental ceramic IPS e.max CAD (Ivoclar Vivadent) have been traditionally used in the anterior region due to the esthetic requirements [8, 11, 12]. LDS consists of 59–63 vol% of a crystalline (crystal size: 1–2 μm, elongated) lithium disilicate (Li_2_Si_2_O_5_) phase, 6–7 vol% lithium orthophosphate (Li_3_PO_4_), and 29–34 vol% of a glass matrix (depending on the crystallization program) [13]. Clinical studies have reported high survival rates for monolithic veneers with more than 98% after four [14] and five years [15, 16], respectively.

Zirconia-reinforced lithium silicate ceramic (ZLS), such as Celtra Duo (Dentsply Sirona) is a ceramic material recommended for similar indications as LDS, such as veneers, inlays, overlays and crowns [7, 17, 18]. ZLS consist of a crystalline mixture of 27 vol% lithium metasilicate (Li_2_SiO_3_), 13 vol% lithium disilicate (Li_2_Si_2_O_5_), 11 vol% lithium orthophosphate (Li_3_PO_4_) with crystallites with a size up to 1 μm, and a glass phase of 49 vol% with dissolved zirconium dioxide (ZrO_2_) fractions of 13 mol% [13]. Survival rates of 97% after two years [19] and 91% in a 5-year follow-up study have been reported for partial crowns [20].

Both ceramics are characterized by comparable flexural strength (three-point bending test; LDS: 377 ± 77 MPa; ZLS: 451 ± 59 MPa) as well as elastic modulus (three-point bending test; LDS: 67 ± 2 GPa; ZLS: 64 ± 4 GPa) and a slightly higher hardness (LDS: 453 ± 17 HV1; ZLS: 595 ± 38 HV1) [17].

Adhesive cementation systems based on resin-based composites are commonly used to bond veneers to tooth structure [21, 22]. Increased strength and resistance of the ceramics due to adhesive luting have been observed for LDS so far [23–25]. The survival rates of veneers are influenced by availability of enamel, preparation technique, the degree of destruction present, tooth functionality, occlusion, parafunctions (bruxism), and tooth vitality [26]. Furthermore, the choice of restorative material, oral hygiene, and prophylaxis measures play a role in longevity [1].

Supragingival professional plaque control, regular calculus removal, and subgingival debridement are essential components for the maintenance of periodontal health [27–30]. Both ultrasonic scaler and powder blasting devices followed by polishing are used for periodontal debridement [31, 32].

For these procedures, previous studies identified an increase in the surface roughness for dental ceramics such as LDS [33, 34] as well as an erosion of the tooth structure [35, 36]. In addition, crack formation in the veneering ceramic due to artificial aging caused by tooth brushing and chewing simulation has been reported [37, 38].

A deepening of the marginal and internal gap of an adhesive interface after simulating thermal loading and prophylactic procedures could also be measured with regard to indirect resin-based composite veneers [39]. However, there is a knowledge gap regarding different silicate ceramic veneers. It is unclear, to what extent the restorative material and the prophylaxis treatment in combination with thermal stress influence the surface, the interface, as well as the internal microstructure with regard to gap formation and cracks.

In this study, extracted premolars were supplied with ceramic veneers made of LDS and ZLS and subjected to artificial aging by means of thermocycling and two different prophylaxis procedures. The objective was to analyze changes in the marginal and internal interface (tooth-veneer) as well as in the surface texture of the ceramic veneers. Two null hypotheses and a further working hypothesis were formulated:

- H_o_(1): The change in the marginal interface due to artificial aging is not dependent on the type of prophylaxis or the choice of veneering material.
- H_o_(2): The surface texture of the veneers is not affected by artificial aging the prophylaxis measures.

*It is also hypothized*, *that the internal interface and the microstructure were not affected with regard to gap and crack formation.*

## Materials and methods

**Fig. 1:**
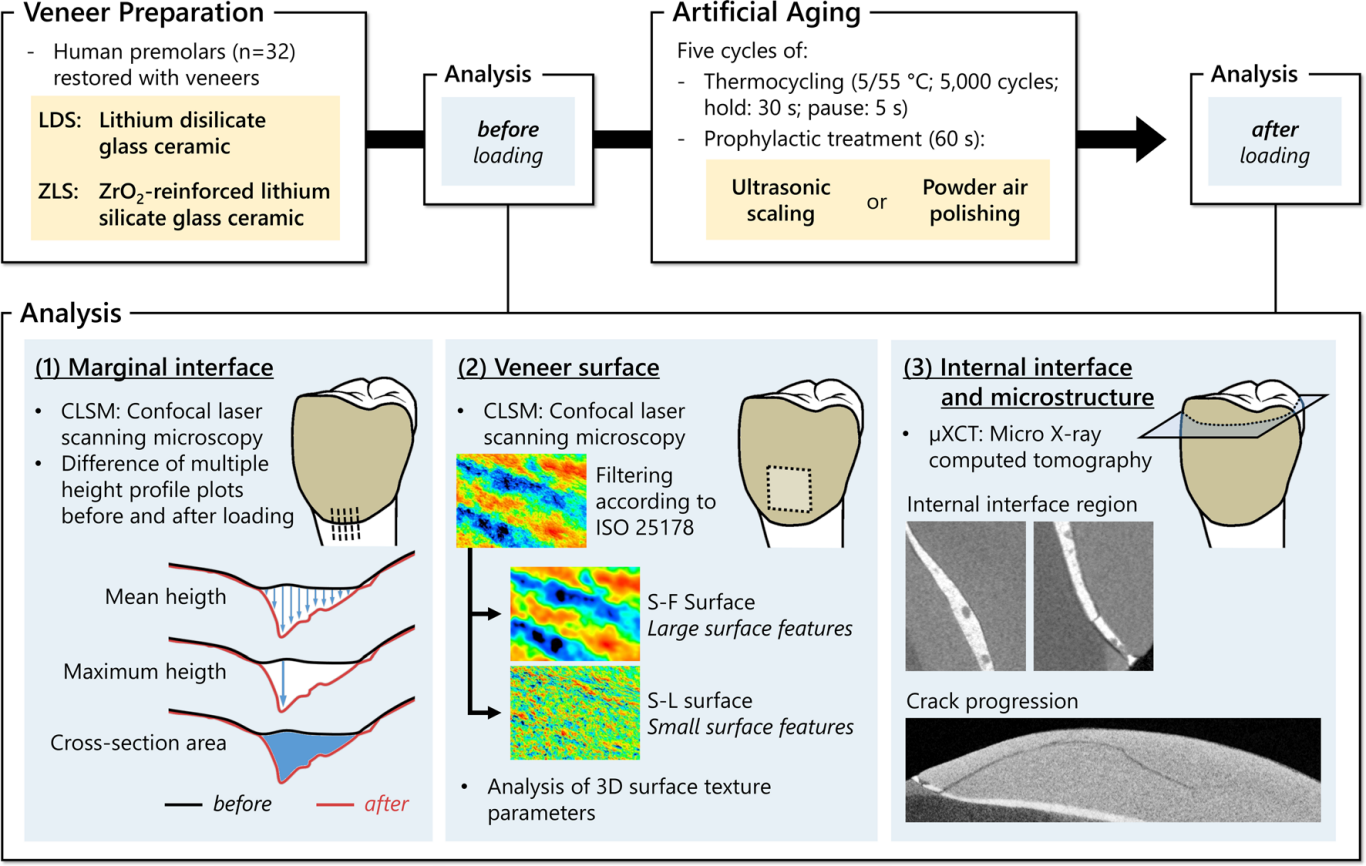
Study design and methodology. The factors of the two-way ANOVA (yellow) were the restorative material (LDS, ZLS) and the prophylaxis treatment (ultrasonic scaling and powder air polishing)

An overview of the study design and methodology for sample preparation, artificial aging and analysis can be found in Fig. 1.

### Sample manufacturing

Thirty-two extracted maxillary and mandibular premolars were selected for this study and divided into two groups (*n* = 16) after visual inspection for fractures, carious lesions, direct or indirect restorations and endodontic pretreatment (approved by the institutional Ethics Committee at the Medical Faculty of the Leipzig University; 286/18-ek). Specimens were stored in freshly prepared 0.5% chloramine-T solution at 4 °C. All teeth were prepared by two experienced dentists (L.A.M., L.U.; supervision: S.H., D.Z.) following the common preparation guidelines for LDS and ZLS. First, orientation grooves of 0.3 mm cervical and 0.4 mm vestibular-coronal were made in the vestibular surfaces of the teeth using a depth marker (supplemental material, Table S1). The orientation grooves were levelled using a congruently shaped diamond bur and finisher, followed by the preparation of an occlusal-vestibular plateau with a reduction of approximately 1.5 mm. The impressions were digitally captured using the Cerec PrimeScan intraoral scanner (Primescan AC 172 with Cerec SW 5 software version 5.1.0.190461, Dentsply Sirona, York, Pennsylvania, USA) and the restorations were digitally designed using the InLab version 19.0 design software (Dentsply Sirona, York, Pennsylvania, USA). The spacer settings were amounted to 80 μm [40]. Sixteen veneers each of lithium disilicate ceramic (LDS) (IPS e.max CAD, Ivoclar Vivadent, Schaan, Liechtenstein) and zirconium oxide-reinforced lithium silicate ceramic (ZLS) (Celtra Duo, Dentsply Sirona, York, Pennsylvania, USA) were fabricated using the inLab MC XL milling machine (Dentsply Sirona, York, Pennsylvania, USA). Any milled edges were removed with a diamond finisher under constant water cooling, followed by crystallization (for LDS) and glaze firing (for LDS and ZLS) according to the manufacturers` protocols (see supplemental material, Table S2). All veneers were etched with 5% hydrofluoric acid (LDS: 20 s; ZLS: 30 s) according to the manufacturers` instructions, rinsed and dried with oil-free air. Conditioning and adhesive placement of the restorations were performed using manufacturer-specific product lines (Table S1) and their respective requirements. Silane was applied for 60 s and teeth were etched in the prepared enamel area with phosphoric acid for 30 s. The adhesive was applied for 20 s followed by gentle drying with oil-free air and light curing for 10 s (Bluephase Style polymerization device, Ivoclar Vivadent, Schaan, Liechtenstein). Adhesive cementation was carried out with a luting composite recommended by the manufacturer (see supplemental material, Table S1). The interface regions were ground and polished with fine diamond burs and ceramic polishers. To ensure reproducible positioning during the prophylactic procedures and analysis, all specimens were embedded in a cold-curing methacrylate-based resin (Technovit 4000, Kulzer, Hanau, Germany) [39]. All materials used are listed in the supplemental material (Table S1).

### Artificial aging

**Fig. 2:**
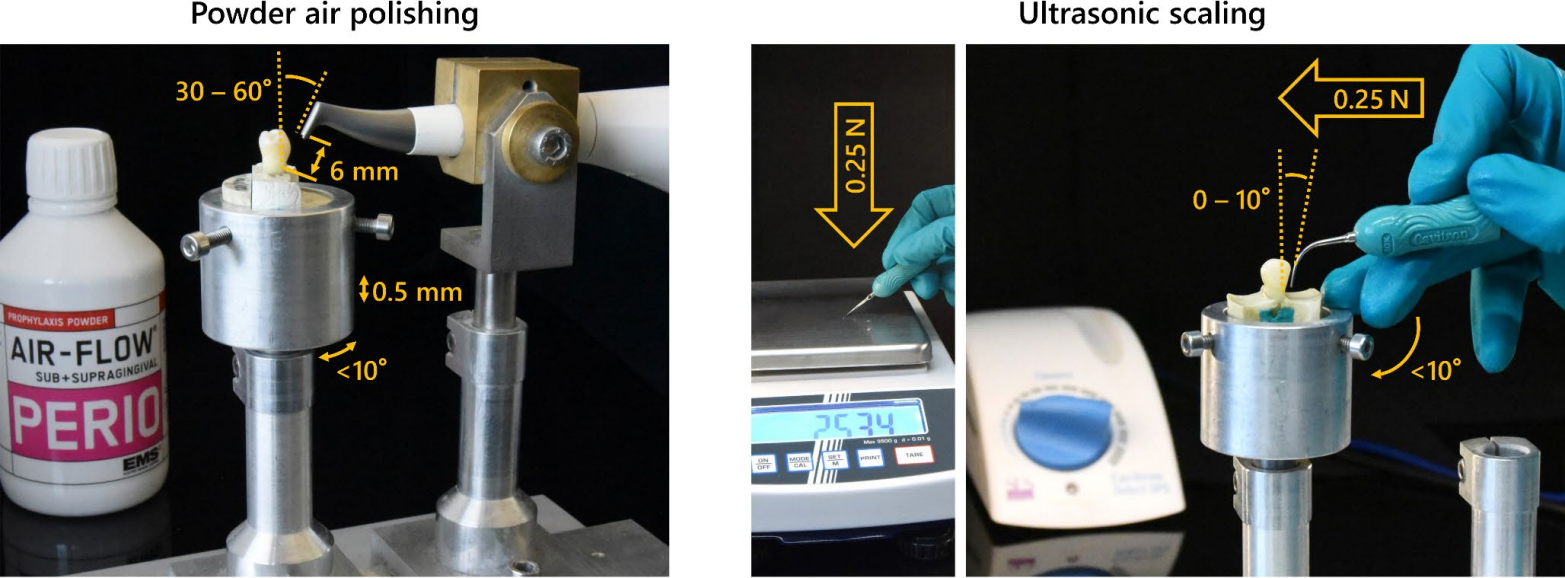
Visualization of the experimental setup for the prophylactic treatments with the corresponding distances, angles, rotational movements and manual calibration of the applied force, left: powder air polishing (Airflowhandy 3.0), right: ultrasonic scaling (Cavitron)

The LDS and ZLS groups were divided into two subgroups (*n* = 8 each) and exposed to thermocycling (Thermocycler THE, SD Mechatronics, Feldkirchen-Westerham, Germany) with 5000 cycles between 5 and 55 °C (dwell time: 30 s; drip-off time: 5 s). Subsequently, two experienced dentists (L.A.M., L.U.; supervision: S.H., D.Z.) performed two different prophylactic treatments. Each subgroup was treated with either powder air polishing or ultrasonic scaling. Powder air polishing was performed using the Airflowhandy 3.0 (with glycine-based Airflow Perio powder, EMS, Nyon, Switzerland; particle size: 25 μm). For a reproducible procedure, a custom-made device was used in which the samples as well as the Airflowhandy 3.0 were mounted in a standardized manner. The distance between the specimens and the exit of the Airflowhandy 3.0 was 6 mm and impacted the surface at an angle of 30–60° within an exposure time of 60 s (Fig. 2). Ultrasonic scaling was carried out by magnetostrictive ultrasonic scaling with the Cavitron FitGrip slim line 30k attachment (Dentsply Sirona, York, Pennsylvania, USA). The vestibular surface of the specimens was subjected to ultrasonic scaling for 60 s at an angle of 0–15° (Fig. 2). The applied contact pressure was approximately 0.25 N and has been calibrated with the aid of a precision scale (PCB 3500–2, Kern, Ballingen, Germany). This sequence of loading by thermocycling and prophylactic treatment was repeated a total of five times for artificial aging (Fig. 1).

### Marginal interface and surface texture

Surface measurements were performed by confocal laser scanning microscopy (CLSM; VK-X1000/X1050, KEYENCE, Osaka, Japan) with a 20x objective (Nikon CF IC EPI Plan 20x; *N* = 0.46; WD = 3.1 mm), a red laser (λ = 661 nm) and an x-y resolution of 2048 × 1536 pixels. Surface measurements were obtained using VK Viewer 1.1.2.174 and analysis was performed with MultiFileAnalyzer 2.1.3.89 (both KEYENCE, Osaka, Japan).

For the analysis of the marginal interface, an F-filter (0.25 mm) was applied. All datasets before and after artificial aging were aligned in position and 15 plot profiles (length: 400 μm; interval: 5 μm) per measurement were placed over the tooth-veneer area, perpendicular to the direction of the marginal interface in the veneer-interface-tooth direction (see Fig. 1, bottom left). Subsequently, the mean and maximum difference as well as the cross-section between the plot line before and after artificial aging in the area of the marginal interface were determined [39].

For the surface texture analysis, an area of the veneer surface was scanned. With appropriate filtering, the S-F (S-filter: 50 μm; F-filter; 0.25 mm; filter type: double gaussian; end effect correction) and S-L (S-filter: 2 μm; F-filter; 0.25 mm; L-filter: 0.05 mm; filter type: double gaussian; end effect correction) surfaces were generated from the data sets. Thus, it was possible to subdivide the analysis into long-scale (S-F surface) or short-scale (S-L surface) surface features. The following surface parameters were analyzed on the basis of the S-F as well as the S-L surface according to ISO 25178-2: *Sa* (arithmetical mean height), *Sq* (root mean square height), *Sdr* (developed interfacial area ratio), *Ssk* (skewness of the height distribution), *Sku* (kurtosis of the height distribution), *Sk* (core height), *Spk* (reduced peak height), *Svk* (reduced valley depth). The selection of parameters is based on the previously published recommendations by Unterschütz et al. (2022) [39].

### Microstructure and internal interface

A total of twelve samples were randomly selected (three for each group) and data sets were acquired using a micro X-ray computed tomography (µXCT, FhG-IKTS-MD, Dresden, Germany). The X-ray microfocus tube (FXE 225.99, YXLON International, Hamburg, Germany) was used in the transmission mode with a tungsten target, a focal spot of 0.6 μm, a copper filter (0.1 mm) and beam energy at 180 kV/150 µA. The radiography was taken with a 2D-detector (2048 × 2048 pitches, CsI; PerkinElmer, Waltham, MA, USA) and reconstructed with Volex v.6.2 (FhG, Dresden, Germany). A resolution of 7 μm was achieved with the selected measurement settings. After processing the raw data with ImageJ 1.47 (National Institutes of Health, Bethesda, USA), all CT datasets before and after artificial aging were visually evaluated according to defects and changes in tooth and veneer surface as well as the internal interface.

### Statistics

All statistical analyses were carried out using IBM SPSS Statistics 29.0.0.0. Normal distribution and homogeneity of variances were assessed with Shapiro-Wilk- and Levene’s test. As no normal distributions were examined for the results of the differences in the surface plot profiles of the marginal interface (mean height, maximum height, cross-section), the data were subjected to an aligned rank transformation and a subsequent two-factor ANOVA (with factors: veneer material LDS/ZLS and prophylactic procedure powder air polishing/ultrasonic scaling). The surface texture parameters (*Sa*, *Sq*, *Sdr*, *Ssk*, *Sku*, *Sk*, *Spk*, *Svk*) before and after artificial aging were subjected to t- or Mann-Whitney-U-test, depending on the occurrence of a normal distribution. The significance level was set at 0.05.

## Results

### Marginal interface and surface texture

**Fig. 3:**
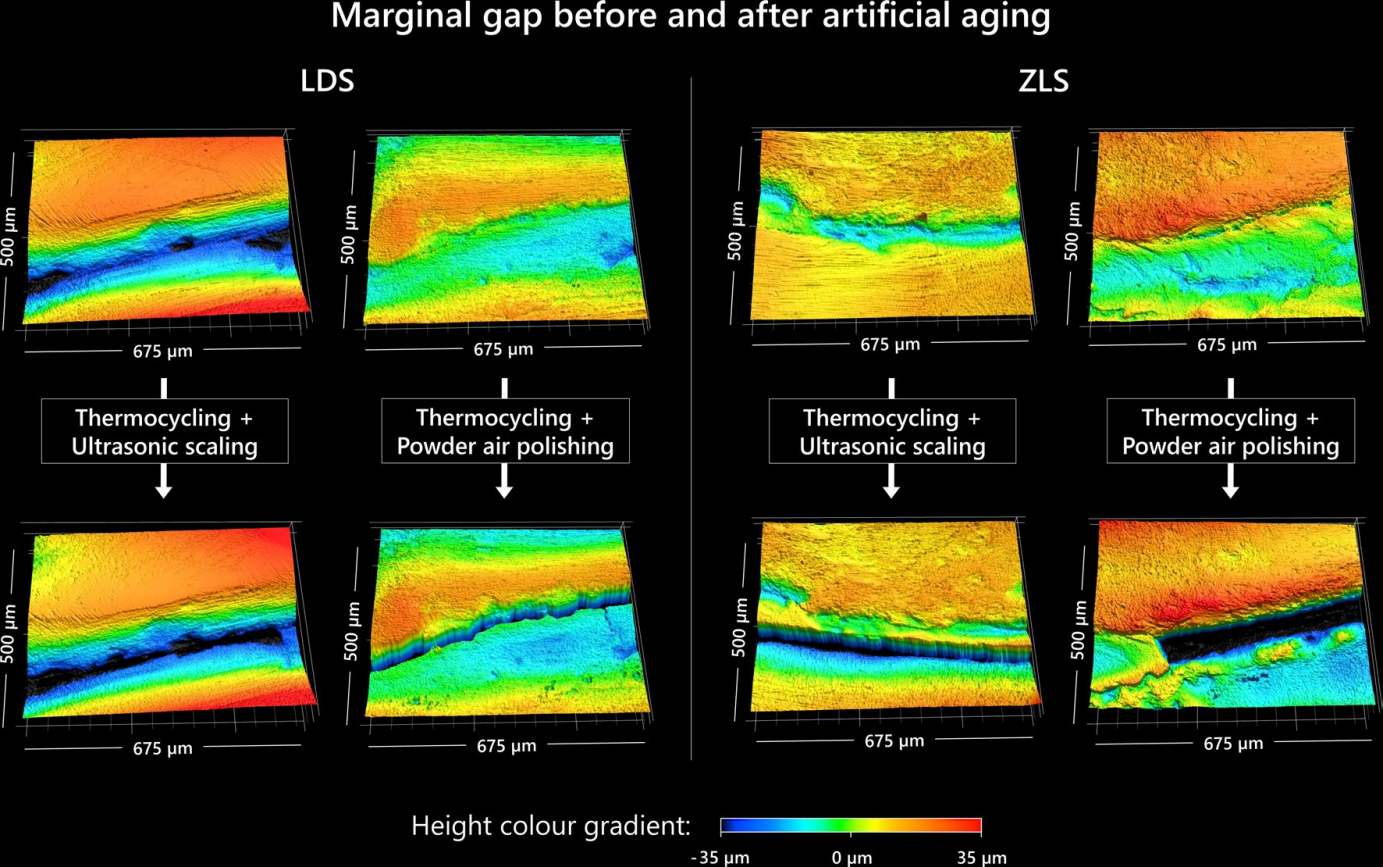
Visualization of the marginal interface (deepened area) of the specimens (veneer: upper area; tooth: lower area) before and after artificial aging

A deepening in the marginal interface, i.e. the area of the luting composite between the tooth and veneer, was observed visually across all groups. The quantification of the gap in terms of mean height, maximum height and cross-section area is shown in Table 1. According to the two-way ANOVA, no significant difference between the type of restorative material (LDS or ZLS) or prophylaxis treatments (ultrasonic scaling or powder air polishing) was detected (mean height: *p* = 0.365; maximum height: *p* = 0.867; cross-section area: *p* = 0.249). Within a group (material and prophylaxis procedure), the marginal interface also exhibited heterogeneous results in the deepening due to artificial aging, which is reflected in a high variance and, thus, a high 95%-confidence interval of the results. An exemplary illustration of the marginal interface tooth-veneer before and after artificial aging is shown in Fig. 3.

**Table 1:** Changes in profile plot lines (median and 95%-CI) across the surface of the marginal interface tooth-veneer

Veneer Material	Prophylaxis treatment	Mean height	Maximum height	Cross-section area
LDS	Ultrasonic scaling	4.51 μm (1.87;12.58)	10.42 μm (4.16;34.94)	256.68 μm² (30.40;1119.54)
	Powder air polishing	4.94 μm (1.21;16.70)	19.55 µm (5.79;38.55)	555.71 μm² (18.69;1606.22)
ZLS	Ultrasonic scaling	15.74 μm (6.44;25.75)	22.71 μm (10.52;41.65)	1525.84 μm² (779.41;2512.83)
	Powder air polishing	8.83 μm (0.82;27.95)	22.49 μm (5.63;49.40)	1109.16 μm² (104.18;3370.88)

The subjective visual assessment of the veneers after artificial aging initially rated both materials ZLS and LDS as rougher in terms of reduced surface gloss. However, after analyzing the surface texture, no significant differences were identified between the surfaces before and after exposure, except for *Ssk* (S-L surface) for LDS when treated with thermocycling and powder air polishing (*p* = 0.038). The results of the surface texture analysis are displayed in the supplemental material (Table S3 and Table S4).

### Microstructure and internal interface

**Fig. 4:**
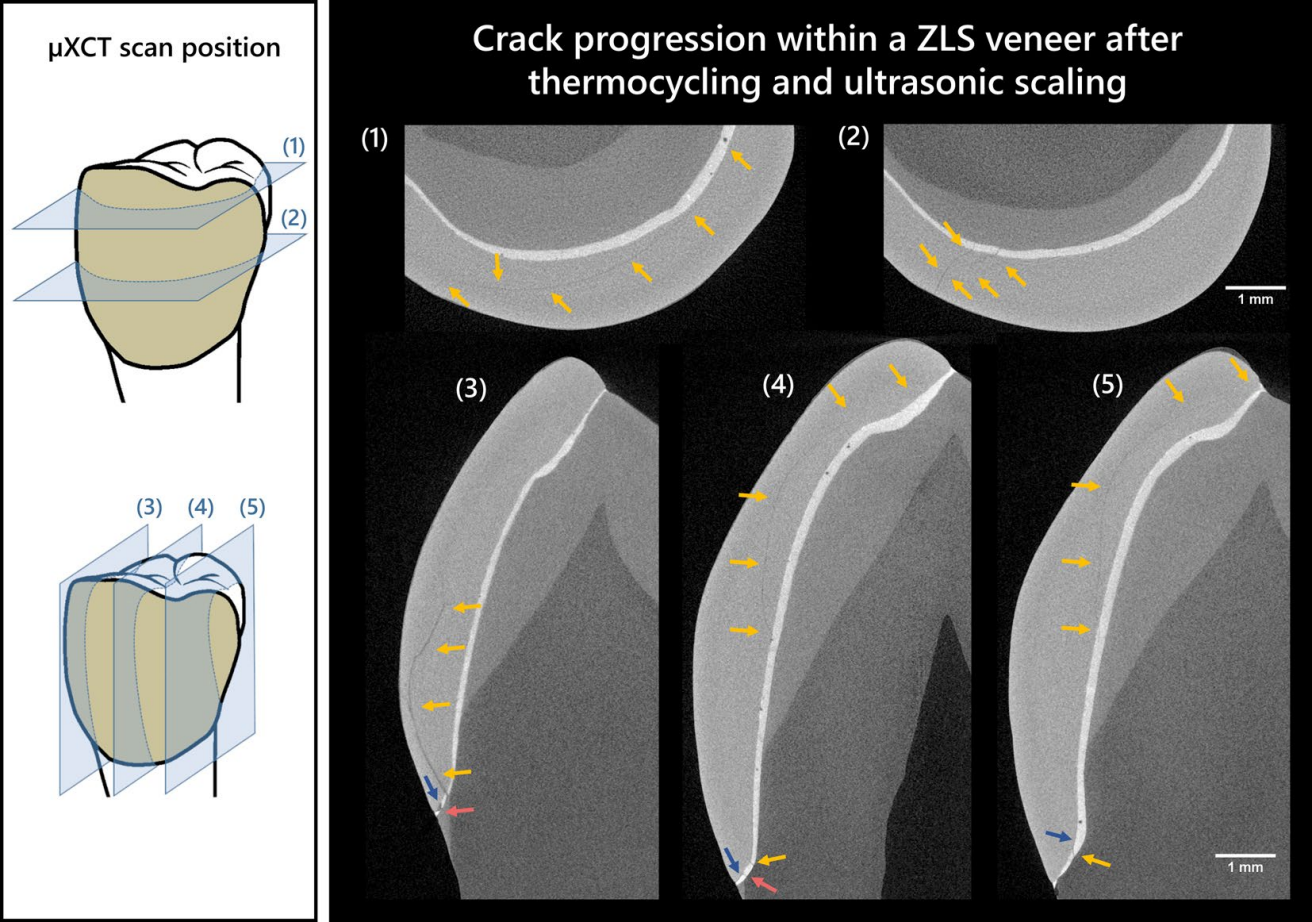
Visualization of the crack progression using µXCT within a zirconia-reinforced lithium silicate veneer (ZLS, ID: CD2) after exposure with thermocycling and ultrasonic scaling in horizontal direction (1, 2; viewed from above) and vertical direction (3, 4, 5; viewed sideways); yellow arrows: Crack formation in the veneer or luting composite; blue arrows: gap formation between veneer and luting composite; red arrows: gap formation between luting composite and tooth

Although no loss of a veneer occurred after the artificial aging, cracks were observed within the veneers of the ZLS group in five out of eight veneer material after exposure to ultrasonic scaling. In three cases, these cracks proceeded diagonally in mesio-distal alignment from the edge and, in two cases, vertically and medially through the veneer. Using µXCT, it was possible to identify a fracture within a ZLS veneer and track its progression (Fig. 4). The crack was primarily located inside the veneer material parallel to the surface and showed minor progression along the internal interface between the veneer/tooth and luting composite. Neither the analyses of the ZLS samples before artificial aging nor the measurements of the LDS samples (before and after) showed any indication of cracks.

**Fig. 5:**
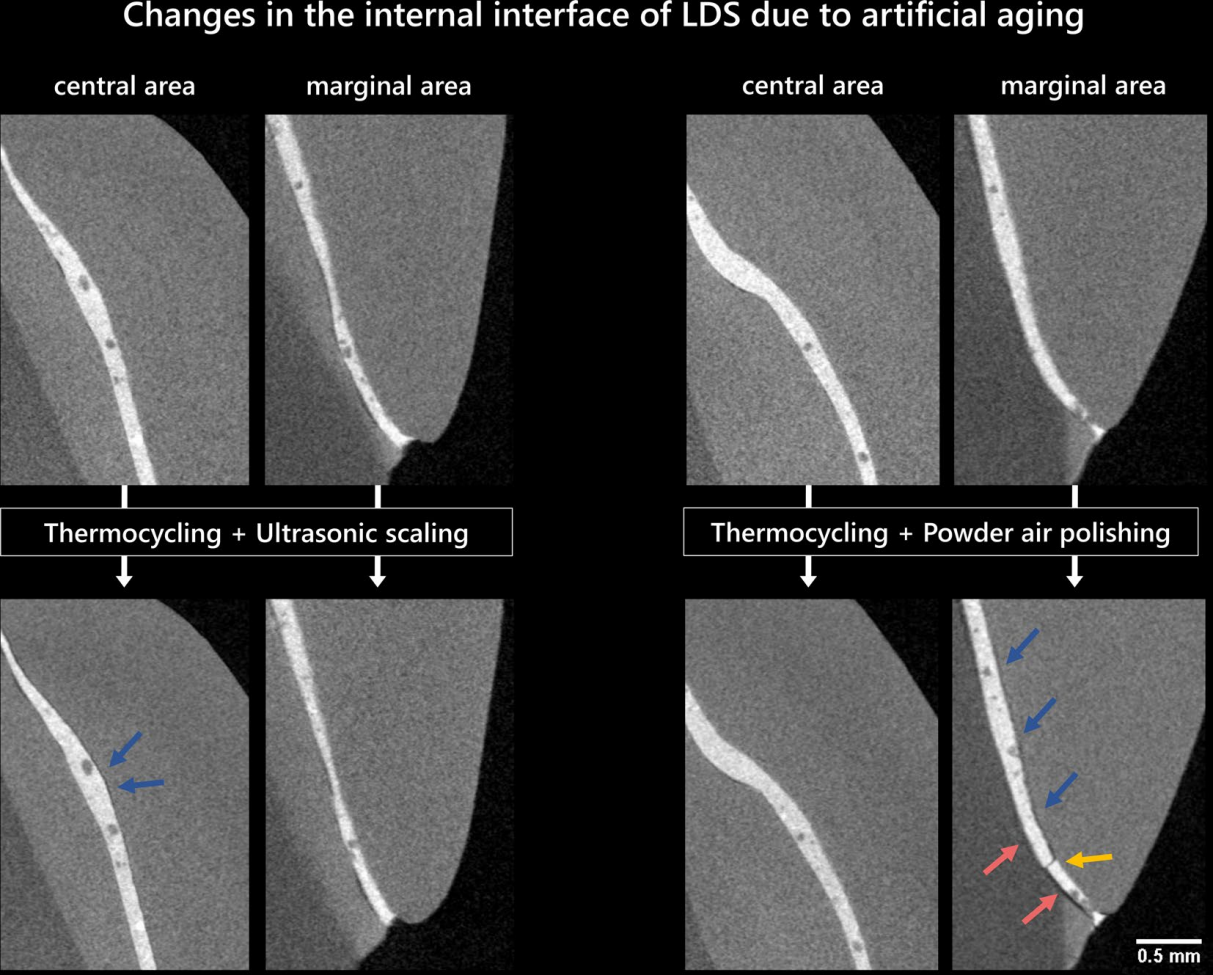
µXCT sectional images of specimens with lithium disilicate veneers (LDS, ID: left: E12/E14, right: EP/EN) in the central and marginal area of the internal interface tooth-veneer before and after exposure with thermocycling and prophylactic treatment; yellow arrows: crack formation within the luting composite; blue arrows: gap formation between veneer and luting composite; red arrows: gap formation between luting composite and tooth

**Fig. 6:**
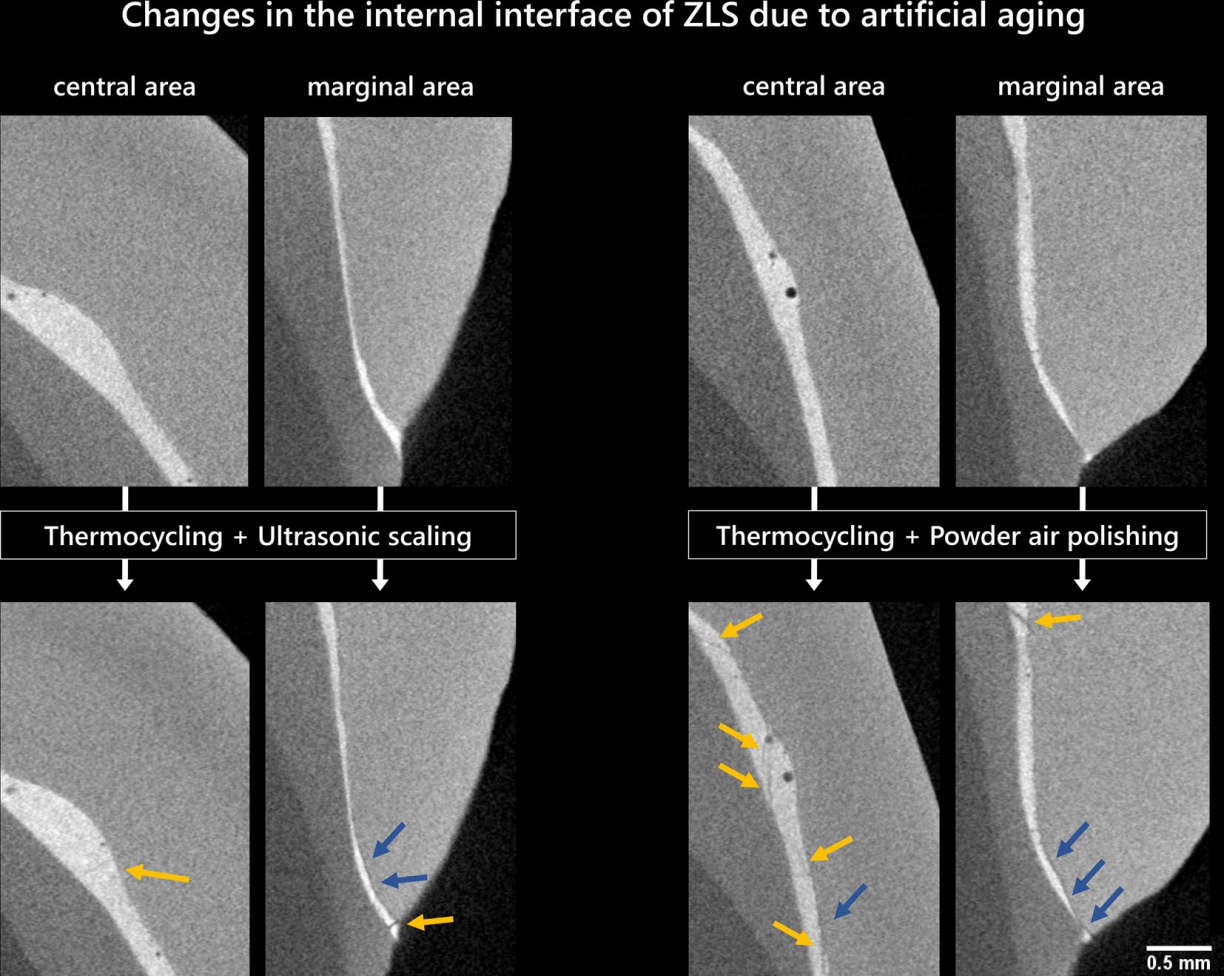
µXCT sectional images of specimens with zirconia-reinforced lithium silicate veneers (ZLS, ID: left: CD3/CD4, right: CD11/CD16) in the central and marginal area of the internal interface tooth-veneer before and after exposure with thermocycling and prophylactic treatment; yellow arrows: crack formation within the luting composite; blue arrows: gap formation between veneer and luting composite

In almost all samples, µXCT measurements revealed gaps in the central region of the internal tooth-veneer interface after preparation prior to artificial aging (Figs. 5 and 6). For the LDS group, one sample each of ultrasonic scaling and powder air polishing showed gap formations primarily between the luting composite and the veneer (Fig. 5). In addition, a crack within the luting composite as well as the formation of gaps between the tooth and the luting composite in the marginal region were observed in the sample exposed to powder air polishing.

In the ZLS group, the formation of gaps exclusively located between the luting composite and the veneer was observed in all test specimens after artificial aging (see yellow arrows in Fig. 6). In addition, cracks were identified in the area of the internal interface for both groups after artificial aging (see yellow arrows in Fig. 6). In one case, ZLS with ultrasonic scaling exposure showed an additional washout effect of the marginal region.

## Discussion

This in vitro study investigated the effect of artificial aging by different prophylaxis treatments in combination with thermocycling on the quality of the marginal interface, surface texture, internal interface, and microstructure of LDS and ZLS ceramic veneers on human premolars.

### Marginal interface and surface texture

Artificial aging affected the marginal interface between the tooth and veneer for all samples of both materials, which can be attributed to the abrasion of the luting composite and an associated deepening of the marginal gap. Since no significant difference was identified between the groups (see two-way ANOVA). Accordingly, the first null hypothesis *“H*_*0*_*(1): The change in the marginal interface due to artificial aging is not dependent on the type of prophylaxis or the choice of veneering material.”* was rejected. It can be derived that the material (LSD or ZLS) or prophylaxis method (ultrasonic scaling or powder air polishing) has no effect on the degree of damage to the marginal tooth-veneer interfaces.

Abrasion as well as a visible increase of roughness of the luting composite in the marginal interface due to ultrasonic scaling was already observed by Andrei et al. (2014) [41]. Similar to the present study, Unterschütz et al. (2022) were able to show that both ultrasonic scaling and powder air polishing result in a deepening of the marginal gap by examining veneers made of polymer-infiltrated ceramic network material and resin-based composite [39]. In the mentioned study, average mean height values of 4.79 μm and maximum height values of 10.39 μm were determined, whereas our investigation with ceramic veneers showed mean heights of 4.51 μm to 15.74 μm and maximum heights of 10.42 μm to 22.71 μm for the marginal interface. These results underline that there is a more pronounced damage to the marginal interface on CAD/CAM ceramics than for veneers made of resin-based composite or polymer infiltrated network material. The depth of damage must also be considered in terms of bond strength. So far, comparable values for the shear bond strength have been determined between CAD/CAM ceramics, resin-based composite or polymer-infiltrated network material and the corresponding luting composites [42, 43]. In addition, Uğur et al. (2023) have shown that a higher shear bond strength can be expected for ceramics compared to a polymer-infiltrated network material in thermally aged samples [44]. Against this background, the greater depth of damage at the marginal interface cannot be attributed to the bonding behavior of the respective materials. The parameters mean height, maximum height and cross-section area showed an overall high variance (reported as 95%-confidence interval), indicating that a heterogeneously pronounced marginal gap is to be expected after treatment with ultrasonic scaling or powder air polishing. This could also be attributed to the locally varying influence of the prophylaxis technique.

In addition to the simulation of a prophylactic treatment, the thermocycling of the restoration is important for estimating the long-term behavior and survival rate. Blunck et al. [45] were able to demonstrate the formation of gaps in the external interface of semi-invasive feldspar ceramic veneers as a result of thermomechanical loading; the specimens in this study were similar to our sample geometry and preparation [48]. Comparable results have been reported by Moon et al. (2021) using LDS ceramic inlays, who identified a deepening of the marginal gap due to the combination of thermocycling and mechanical loading with chewing simulation [46]. With the additional influence of regular prophylactic treatment, a deterioration of the marginal tooth-veneer interface in the life cycle of a restoration is to be expected, which should be taken into account in clinical practice.

A change in the surface texture of the ceramic veneers could only be observed for LDS after exposure to powder air polishing in the skewness of the surface distribution (*Ssk*) on a short-scale level (S-L surface). Therefore, the second null hypothesis “*H*_*0*_*(2):The surface texture of the veneers is not affected by artificial aging the prophylaxis measures”* was partially rejected.

Since there were no statistically significant changes in other surface texture parameters and the resulting p-value of *p* = 0.038 for the change of *Ssk* is close to the specified significance level of α = 0.050, we doubt a change in surface texture under the given analysis conditions. In contrast, a previous study with veneers fabricated from polymer-infiltrated ceramic network material using thermocycling in combination with ultrasonic scaling demonstrated a change in surface texture based on several parameters (S-L surface: increasing *Sdr*, *Sku*, *Svk*; decreasing *Spk*) [39]. Our results show that, compared to resin based composites (62 ± 10 HV1) as well as polymer-infiltrated ceramic (157 ± 14 HV1), the use of ceramics (LDS: 453 ± 17 HV1; ZLS: 595 ± 38 HV1) as a restorative material may result in greater resistance of the veneer surface to prophylactic treatment due to the higher hardness of the surface [17]. Increased biofilm accumulation on the lithium silicate ceramics is not to be expected after repeated prophylaxis and aging. However, a greater damage within the marginal tooth-veneer interface should be considered.

### Microstructure and internal interface

µXCT investigation revealed changes in the interface regions of both ceramics LDS and ZLS. For the samples of the LDS group, gaps were observed between the luting composite and the veneer for both prophylactic treatments in combination with thermal loading. For the samples of the ZLS group, these gaps in the internal interface were qualitatively larger than for the LDS specimens. In addition, five out of eight samples within the ZLS veneers exhibited cracks within the ceramic material. At this point, we have to withdraw our hypothesis, that the internal interface and the microstructure were not affected with regard to gap and crack formation.

In addition to the mechanical loading caused by ultrasonic scaling or powder air polishing, thermocycling with a temperature gradient of 5 and 55 °C can cause high mechanical constraint stresses, especially for specimens fabricated from brittle materials with low thickness, if the coefficient of thermal expansion (CTE) differs from the luting composite. It should be noted that our study design includes a total of 25,000 thermal cycles over the entire course of artificial aging. This implies an extensive stress, especially as the 5–55 °C temperature interval is widely used in dental materials science in vitro testings for the simulation in clinical practice, but represents a high thermal load and does not necessarily correspond to a clinical environment [47].

In general, for most dental materials, CTEs are not well known for the temperature range of 5–55 °C. The materials used in the current study feature different CTEs, with 10.5 10^− 6^/K (100–400 °C) for LDS, 11.8 10^− 6^/K (25–500 °C) for ZLS (both manufacturer’s specifications), and 22–45 10^− 6^/K (26–75 °C) for luting composites [47–49]. In contrast, CTE of dentin and enamel are 11 10^− 6^/K and 17 10^− 6^/K (both 10–80 °C), respectively [48]. In addition, shrinkage of the luting composite is to be expected. These effects can lead to internal stresses and cause microcracks in the interface or the restorative material itself [50]. Compared to veneers made of resin-based composite or polymer-infiltrated ceramic network materials [39], the more brittle ceramics (elastic modulus by three-point bending test: 12–22 GPa vs. 61–67 GPa) exhibited a higher potential for splitting and cracking of the internal interface due to the stresses investigated [17]. Damage to the restoration due to cracks and/or microleakage and a reduction in the bond strength of ZLS have already been observed in previous studies [51, 52]. Also Alnakib and Alsaady (2021) reported a higher microleakage of veneers made of ZLS compared to LDS [53].

Since the orientation of the crack pattern in ZLS veneers is parallel rather than perpendicular to the surface, it is more indicative of constraining stresses in the structure than of external mechanical stresses. It remains unclear, why ZLS but not LSD showed cracks despite its higher strength (ZLS: 451 MPa vs. LDS: 377 MPa) and low modulus of elasticity (ZLS: 64 GPa vs. LDS: 67 GPa). The CTEs probably differ in the temperature range 5–55 °C, taking into account the phase composition, glass fraction, and specific crystallite geometry. Due to the different CTEs, thermocycling would lead to temperature gradients in the material and thus constraining stresses in the microstructure, which exceed the strength in the case of ZLS.

Clinical studies have reported survival rates of 99–100% at four to five years for LDS veneers [14, 15]. In addition, survival rates of 99% after four years were documented for LDS single crown restorations [14] and 97% after seven years for LDS partial coverage crowns [54]. A clinical study by Rinke et al. showed a 97% survival rate for ZLS partial coverage crowns after two years and a 91% survival rate in the follow-up study after five years [19, 20]. Comparable studies with survival rates for ZLS veneers are currently not available. Based on our results, it can be assessed that – despite the high survival rates of partial crowns – ZLS veneers may have a higher potential for intra-material cracking and a higher failure could be expected in the long term than for LDS veneers in the clinical environment.

### Limitations

Due to the geometry of the natural teeth and despite a thorough and carefully followed preparation regime, exposure of dentin during preparation and a resulting influence on adhesion cannot be excluded. Veneers bonded in the enamel showed a significantly higher survival rate than those bonded in the dentin [55, 56]. In addition, differences in the teeth across the population should be considered, and the number of samples should be adjusted for future studies according to the region of interest (e.g. marginal or internal interface). With regard to stress, no mechanical simulation of chewing forces was used for artificial ageing in addition to prophylactic treatment. Chewing forces may promote cracking and fracture and should be simulated for a more accurate estimation of the survival rate of veneers from in vitro tests [37, 57]. Particularly for the use of veneers in the anterior region, tensile and compressive forces caused by the complex chewing and tearing movement should be taken into account [58]. Therefore, further studies are recommended to simulate these complex masticatory forces in the anterior region in order to provide a more precise assessment of the survival rate and clinical environment.

The veneers were used within the guidelines of the manufacturer’s recommendations in order to align the in vitro study with clinical application and draw tangible conclusions for clinical practice. Thus, this study not only highlights a difference in the ceramic materials LDS and ZLS, but also in their respective processing and use as recommended. The differences must therefore also be placed in the context of the respective preparation protocol (e.g. etching time) and the luting composite applied. Further research into the influence of preparation-related factors is recommended. A comparison of the two prophylaxis methods of powder air polishing and ultrasonic scaling is also only possible to a limited extent due to the different reproducibility of the application. A higher peak load due to the less standardizable ultrasonic scaling should be taken into account when examining the cracks within the veneer.

Regarding the analytical methods, it should be emphasized that µXCT measurements can only detect gaps and pores above the resolution limit (in our case: >7 μm). In addition, the esthetic appearance was not evaluated. Further investigations in combination with the analysis of the optical properties and µXCT are recommended for the influence of adhesive bonding, which has already been used as a promising approach for the analysis of the internal interface. The limitation of the surface roughness investigation in the present study is caused by the geometry of the specimens. Further investigation of the materials in plane format (slices) is recommended in order to achieve higher magnifications (CLSM: >20x objective) and greater precision. Additionally, plane specimens would allow an additional reproducible analysis of optical properties such as gloss, translucency, or color change.

## Conclusions

Both ultrasonic scaling and powder air polishing in combination with thermal loading resulted in abrasion of the luting composite and deepening of the marginal tooth-veneer interface of natural premolars restored with ceramic veneers, regardless of the chosen ceramic material. No changes in surface roughness of LDS or ZLS veneers were observed under the given test conditions. After artificial aging, the internal interface showed defects between luting composite and ceramic veneers. ZLS veneers showed larger gaps and partial cracks parallel to the ceramic surface after artificial aging using ultrasonic scaling. With regard to artificial aging, ZLS also showed a higher failure rate, from which a better clinical performance can be assumed for LDS. Accordingly, the use of ultrasonic scaling for veneers made of ZLS should be considered critical in everyday clinical practice, as damage can occur within the material and the internal interface. In the prophylactic measure of LDS veneers, no special consideration needs to be given to the choice of treatment.

The investigative approach of surface measurement using CLSM combined with microstructural analysis using µXCT provides a comprehensive and non-destructive view of the damage mechanisms of the marginal and internal interface.

## Electronic supplementary material

Below is the link to the electronic supplementary material.

Supplementary Material 1

## Data Availability

No datasets were generated or analysed during the current study.
