# Supplementary material for: Effect of powder air polishing and ultrasonic scaling on the marginal and internal interface (tooth-veneer) of ceramic veneers: an in vitro study
Source: Clin Oral Investig. 2024 Nov 26;28(12):655. doi: 10.1007/s00784-024-06046-x (PMC11599320; doi:10.1007/s00784-024-06046-x)
Supplement: Supplementary file 1 — Supplementary Material 1 [file 784_2024_6046_MOESM1_ESM.docx]

**Effect of powder air polishing and ultrasonic scaling on the marginal and internal interface (tooth-veneer) of ceramic veneers: An in vitro study**

# *– Supplemental material –*

*Table S1: Materials and preparations tools used in this study.*

|  | **Material or Tool** | **Manufacturer** | **LOT** |
| --- | --- | --- | --- |
| **ZLS** | Celtra Duo HT A2, C14 | Dentsply Sirona, York, Pennsylvania, USA | 16005516, 16005517  16008156, 16010642 |
|  | DeTrey Conditioner 36 |  | 2007000136 |
|  | Calibra Silane Coupling Agent |  | 00050413 |
|  | Calibra Veneer Esthetic Resin Cement, shade light |  | 00050685 |
|  | Prime and Bond XP |  | 2002000057 |
| **LDS** | IPS e.max CAD HT A2, C14 | Ivoclar Vivadent, Schaan, Liechtenstein | X39494, Z006FZ |
|  | Monobond Plus |  | Z004FP |
|  | Total Etch (37% phosphoric acid) |  | Z00748 |
|  | Adhese Universal VivaPen |  | Z00466 |
|  | Variolink LC esthetic light |  | Y39814 |
| **ZLS and LDS** | IPS Ceramic Etching Gel 5% | Ivoclar Vivadent, Schaan, Liechtenstein | X39271 |
|  | IPS Object Fix Putty |  | Y30754 |
|  | Chloramine T trihydrate ACS reagent, 98% | Sigma-Aldrich, St. Louis, USA | STBF3027V |
|  | Technovit 4000 Powder | Heraeus-Kulzer, Hanau, Germany | R010028 |
|  | Technovit Sirup 1 |  | R010024 |
|  | Technovit Sirup 2 |  | R010023 |
|  | Prep Marker Set 4663 | Komet Dental, Lemgo, Germany | 00005039 |
|  | Depth marker 868B 314 018 |  | 191837 |
|  | Diamond bur 8868 314 016 |  | 199657 |
|  | Diamond finisher 8868 314 016 |  | 00104703 |
|  | Diamond finisher 379EF 314 023 |  | 00072677 |
|  | Cylinder Pointed Bur 20 | Dentsply Sirona, York, Pennsylvania, USA | M49601 |
|  | Step Bur 20 |  | M53879 |
|  | Step Bur 20 |  | M37752 |
|  | Step Bur 12 S |  | M37816 |
|  | Cylinder Pointed Bur 12 S |  | M72231 |
|  | Cylinder Pointed Bur 12 S |  | M83130 |
|  | Airflow Pulver Perio (25 µm) | EMS Dental, Nyon, Switzerland | 1909163 |

*Table S2: Sintering parameters for the CAD/CAM ceramics.*

| **LDS (IPS e.max CAD)** | | **ZLS (Celtra Duo)** | |
| --- | --- | --- | --- |
| **Crystallization and Glaze Firing**  **Programat CS** | | **Glaze Firing**  **Vita Vacumat 40T** | |
| Entry time | 6:00 min | Start temperature | 500 °C |
| Entry temperature | 403 °C | Preheating | 4:07 min |
| Firing temperature | 820 °C | Final temperature | 820 °C |
| Holding time | 0:10 min | Holding time | 1:00 min |
| Firing temperature | 840 °C | Long-term cooling | 3:00 min |
| Holding time | 7:00 min |  | |
| Long-term cooling | 700 °C |  |  |
| Vacuum 1 | 550 °C, 820 °C |  |  |
| Vacuum 2 | 820 °C, 840 °C |  |  |

*Table S3: Surface texture parameters (mean and SD) for processed S-F and S-L surfaces of lithium disilicate glass ceramic (LDS) before and after loading by thermocycling and prophylactic treatment (powder air polishing or ultrasonic scaling).*

| **LDS** |  |  | ***Sa* (µm)** | ***Sq* (µm)** | ***Sdr* (%)** | ***Ssk*** | ***Sku*** | ***Sk* (µm)** | ***Spk* (µm)** | ***Svk* (µm)** |
| --- | --- | --- | --- | --- | --- | --- | --- | --- | --- | --- |
|  |  |  |  |  |  |  |  |  |  |  |
| **Powder air polishing** | **S-F  Surface** | *before* | 0.66 ± 0.33 | 0.84 ± 0.42 | 0.11 ± 0.11 | 0.85 ± 0.66 | 3.80 ± 1.24 | 1.65 ± 1.10 | 0.77 ± 0.41 | 0.78 ± 0.35 |
|  |  | *after* | 0.64 ± 0.31 | 0.82 ± 0.39 | 0.10 ± 0.09 | 0.74 ± 0.87 | 4.18 ± 2.46 | 1.59 ± 1.01 | 0.78 ± 0.43 | 0.78 ± 0.31 |
|  |  | *p-value* | 0.721 | 0.798 | 0.878 | 1.000 | 0.959 | 0.959 | 0.959 | 0.959 |
|  |  |  |  |  |  |  |  |  |  |  |
|  | **S-L  Surface** | *before* | 0.73 ± 0.20 | 0.96 ± 0.28 | 14.90 ± 7.05 | -0.95 ± 0.08 | 4.85 ± 0.97 | 1.99 ± 0.48 | 1.07 ± 0.36 | 1.09 ± 0.41 |
|  |  | *after* | 0.70 ± 0.24 | 0.91 ± 0.30 | 13.02 ± 7.82 | -1.03 ± 0.06 | 4.28 ± 0.69 | 1.99 ± 0.70 | 0.94 ± 0.28 | 1.01 ± 0.29 |
|  |  | *p-value* | 0.645 | 0.574 | 0.234 | **0.038** | 0.234 | 0.645 | 0.328 | 0.721 |
|  |  |  |  |  |  |  |  |  |  |  |
|  |  |  |  |  |  |  |  |  |  |  |
| **Ultrasonic scaling** | **S-F  Surface** | *before* | 0.55 ± 0.17 | 0.69 ± 0.22 | 0.06 ± 0.02 | 1.23 ± 0.44 | 3.23 ± 0.56 | 1.27 ± 0.31 | 0.69 ± 0.34 | 0.65 ± 0.16 |
|  |  | *after* | 0.52 ± 0.14 | 0.65 ± 0.19 | 0.05 ± 0.03 | 1.03 ± 0.38 | 3.01 ± 0.76 | 1.12 ± 0.28 | 0.56 ± 0.28 | 0.73 ± 0.30 |
|  |  | *p-value* | 0.645 | 0.574 | 0.878 | 0.195 | 0.195 | 0.234 | 0.382 | 1.000 |
|  |  |  |  |  |  |  |  |  |  |  |
|  | **S-L  Surface** | *before* | 0.62 ± 0.15 | 0.86 ± 0.16 | 13.21 ± 7.42 | -1.39 ± 0.69 | 8.49 ± 5.29 | 1.51 ± 0.54 | 0.99 ± 0.27 | 1.15 ± 0.11 |
|  |  | *after* | 0.59 ± 0.11 | 0.81 ± 0.12 | 10.71 ± 4.07 | -1.36 ± 0.84 | 9.01 ± 9.57 | 1.43 ± 0.43 | 0.95 ± 0.20 | 1.08 ± 0.21 |
|  |  | *p-value* | 0.798 | 0.645 | 0.798 | 0.959 | 0.574 | 0.645 | 0.959 | 0.442 |

*Table S4: Surface texture parameters (mean and SD) for processed S-F and S-L surfaces of zirconia-reinforced lithium silicate glass ceramic (ZLS) before and after loading by thermocycling and prophylactic treatment (powder air polishing or ultrasonic scaling).*

| **ZLS** |  |  | ***Sa* (µm)** | ***Sq* (µm)** | ***Sdr* (%)** | ***Ssk*** | ***Sku*** | ***Sk* (µm)** | ***Spk* (µm)** | ***Svk* (µm)** |
| --- | --- | --- | --- | --- | --- | --- | --- | --- | --- | --- |
|  |  |  |  |  |  |  |  |  |  |  |
| **Powder air polishing** | **S-F  Surface** | *before* | 0.58 ± 0.12 | 0.73 ± 0.13 | 0.09 ± 0.05 | 1.06 ± 0.87 | 5.79 ± 6.89 | 1.31 ± 0.31 | 0.57 ± 0.18 | 0.83 ± 0.17 |
|  |  | *after* | 0.57 ± 0.12 | 0.70 ± 0.12 | 0.07 ± 0.03 | 0.80 ± 0.59 | 3.11 ± 1.52 | 1.26 ± 0.33 | 0.43 ± 0.12 | 0.79 ± 0.25 |
|  |  | *p-value* | 0.798 | 0.645 | 0.382 | 0.721 | 0.442 | 0.798 | 0.130 | 0.721 |
|  |  |  |  |  |  |  |  |  |  |  |
|  | **S-L  Surface** | *before* | 0.76 ± 0.18 | 1.05 ± 0.23 | 18.34 ± 7.76 | -1.08 ± 0.47 | 6.01 ± 1.51 | 1.92 ± 0.52 | 1.25 ± 0.41 | 1.39 ± 0.17 |
|  |  | *after* | 0.69 ± 0.12 | 0.93 ± 0.15 | 15.52 ± 5.06 | -1.02 ± 0.28 | 5.21 ± 0.98 | 1.80 ± 0.35 | 1.09 ± 0.24 | 1.18 ± 0.17 |
|  |  | *p-value* | 0.574 | 0.328 | 0.721 | 0.798 | 0.328 | 0.798 | 0.721 | 0.065 |
|  |  |  |  |  |  |  |  |  |  |  |
|  |  |  |  |  |  |  |  |  |  |  |
| **Ultrasonic scaling** | **S-F  Surface** | *before* | 0.58 ± 0.10 | 0.76 ± 0.19 | 0.10 ± 0.07 | 0.83 ± 0.71 | 5.70 ± 4.90 | 1.25 ± 0.27 | 0.79 ± 0.52 | 0.98 ± 0.34 |
|  |  | *after* | 0.54 ± 0.08 | 0.73 ± 0.19 | 0.10 ± 0.09 | 0.69 ± 0.66 | 11.06 ± 19.87 | 1.16 ± 0.20 | 0.71 ± 0.43 | 0.86 ± 0.35 |
|  |  | *p-value* | 0.442 | 0.382 | 0.574 | 0.645 | 0.645 | 0.382 | 0.574 | 0.505 |
|  |  |  |  |  |  |  |  |  |  |  |
|  | **S-L  Surface** | *before* | 0.75 ± 0.15 | 1.01 ± 0.19 | 17.12 ± 5.92 | -1.14 ± 0.28 | 5.43 ± 0.75 | 1.89 ± 0.48 | 1.16 ± 0.24 | 1.36 ± 0.24 |
|  |  | *after* | 0.72 ± 0.09 | 0.98 ± 0.09 | 16.21 ± 5.11 | -0.90 ± 0.36 | 5.51 ± 0.80 | 1.75 ± 0.35 | 1.25 ± 0.21 | 1.27 ± 0.13 |
|  |  | *p-value* | 0.382 | 0.442 | 0.878 | 0.161 | 0.959 | 0.382 | 0.382 | 0.234 |
